# Multiscale Photoacoustic Tomography of a Genetically Encoded Near‐Infrared FRET Biosensor

**DOI:** 10.1002/advs.202102474

**Published:** 2021-09-17

**Authors:** Lei Li, Hsun‐Chia Hsu, Vladislav V. Verkhusha, Lihong V. Wang, Daria M. Shcherbakova

**Affiliations:** ^1^ Caltech Optical Imaging Laboratory, Andrew and Peggy Cherng Department of Medical Engineering and Department of Electrical Engineering California Institute of Technology Pasadena CA 91125 USA; ^2^ Medicum, Faculty of Medicine University of Helsinki Helsinki 00290 Finland; ^3^ Department of Anatomy and Structural Biology and Gruss‐Lipper Biophotonics Center Albert Einstein College of Medicine Bronx NY 10461 USA; ^4^ Science Center for Genetics and Life Sciences Sirius University of Science and Technology Sochi 354340 Russia

**Keywords:** bacterial phytochrome, biosensor, BphP, FRET, photoacoustic computed tomography (PACT), photoacoustic microscopy (PAM), photoacoustic microscopy

## Abstract

Photoacoustic tomography (PAT) with genetically encoded near‐infrared probes enables visualization of specific cell populations in vivo at high resolution deeply in biological tissues. However, because of a lack of proper probes, PAT of cellular dynamics remains unexplored. Here, the authors report a near‐infrared Forster resonance energy transfer (FRET) biosensor based on a miRFP670‐iRFP720 pair of the near‐infrared fluorescent proteins, which enables dynamic functional imaging of active biological processes in deep tissues. By photoacoustically detecting the changes in the optical absorption of the miRFP670 FRET‐donor, they monitored cell apoptosis in deep tissue at high spatiotemporal resolution using PAT. Specifically, they detected apoptosis in single cells at a resolution of ≈3 µm in a mouse ear tumor, and in deep brain tumors (>3 mm beneath the scalp) of living mice at a spatial resolution of ≈150 µm with a 20 Hz frame rate. These results open the way for high‐resolution photoacoustic imaging of dynamic biological processes in deep tissues using NIR biosensors and PAT.

## Graphical abstract

Photoacoustic tomography with genetically encoded probes allows for the imaging of specific cell populations in vivo. The authors report near‐infrared biosensor enabling dynamic functional imaging of active biological processes in deep tissues. The author also detect apoptosis in single cells and in deep‐brain tumors of living mice. The results pave the way for high‐resolution photoacoustic imaging of biological processes in deep tissues.

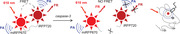

## 1 Introduction

Photoacoustic (PA) tomography (PAT) breaks the optical diffusion limit by acoustically detecting optical absorption contrast.^[^ ^1^ ^]^ Acoustic waves are orders of magnitude less scattered in biological tissues, providing PAT far better spatial resolution than pure optical imaging in deep tissues (>2 mm).^,[^ ^2^, ^3^ ^]^ PAT is sensitive to the optical absorption of molecules. It is powerful at detecting both endogenous molecules, such as hemoglobin, cytochromes, deoxyribonucleic acid/ribonucleic acid (DNA/RNA), and melanin, and exogenous probes, such as organic dyes, nanoparticles, and genetically encoded chromophore‐containing proteins.^[^ ^4^, ^5^, ^6^, ^7^, ^8^, ^9^, ^10^, ^11^, ^12^, ^13^, ^14^ ^]^

The use of genetically encoded bacteriophytochrome (BphP) based near‐infrared (NIR) fluorescent proteins (FPs) as contrast molecules in PAT provides the most advanced technology for deep‐tissue visualization of molecules and cells at high spatial resolution in vivo.^[^ ^15^, ^16^, ^17^, ^18^, ^19^, ^20^, ^21^ ^]^ The BphP‐based molecules with absorption peaks at 640–780 nm can be clearly distinguished from hemoglobin and provide the best sensitivity for in vivo visualization of cell populations.^[^ ^22^ ^]^ As chromophores, they incorporate biliverdin, which is abundant in eukaryotic cells as a product of heme metabolism.^[^ ^23^ ^]^ While PAT with BphP‐based molecules is rapidly developing,^[^ ^17^, ^19^, ^24^ ^]^ so far, it has been applied to structural imaging, leaving dynamic molecular processes largely unexplored.

Biological phenomena result from physico‐chemical processes of molecular binding, association, conformational change, and catalysis.^[^ ^25^ ^]^ To visualize dynamic processes, it is necessary to elucidate the functional states of the constituent molecules at different time points. Förster resonance energy transfer (FRET), uniquely sensitive to molecular conformation, association, and separation in the 1–10 nm range, can resolve molecular interactions and conformations.^[^ ^26^ ^]^ Naturally, fluorescence microscopy is suited for FRET imaging, which has provided valuable information for biomedical research. Multiphoton microscopy has been widely used for FRET imaging with extended penetration depth.^[^ ^27^, ^28^, ^29^, ^30^, ^31^, ^32^ ^]^ However, strong optical scattering in biological tissue impedes high spatial resolution fluorescence imaging of FRET at depths beyond 2 mm. Photoacoustic FRET imaging of dyes^[^ ^33^ ^]^ and FPs of green fluorescent protein (GFP) family‐based biosensors^[^ ^26^ ^]^ was shown in model systems with purified molecules in vitro. However, the use of FRET sensors absorbing in the blue/green light range seems to be incompatible with PAT imaging of deep tissues in vivo, because the strong optical absorption and scattering from endogenous molecules fundamentally limit blue/green light penetration.^[^ ^34^ ^]^

Recently, following the development of spectrally distinct NIR FPs, such as those of (m)iRFP series of proteins,^[^ ^35^, ^36^, ^37^, ^38^, ^39^ ^]^ genetically encoded NIR FRET biosensors became available. NIR FRET biosensors for GTPase,^[^ ^37^ ^]^ protein kinases,^[^ ^37^, ^40^ ^]^ and calcium dynamics^[^ ^41^ ^]^ allowed multiplexing with visible FPs for monitoring several processes in single cells.^[^ ^42^ ^]^ NIR biosensors also can be combined with blue‐light controlled optogenetic tools for cross‐talk free all‐optical control and readout.^[^ ^42^ ^]^ NIR spectrum of these biosensors is obviously advantageous for their deep and sensitive imaging in living cells and live animals, because of deeper light penetration, less scattering, and minimal autofluorescence.^[^ ^43^ ^]^

In this work, we applied a genetically encoded NIR FRET biosensor as a probe in PAT. We performed multiscale imaging of biological processes based on FRET in vivo for the first time. We investigated two incarnations of PAT, photoacoustic microscopy (PAM)^[^ ^44^ ^]^ for a shallow tumor in a mouse ear at optical resolution and photoacoustic computed tomography (PACT)^[^ ^9^ ^]^ for a deep‐seated tumor in the brain at a resolution limited by acoustic diffraction, allowing imaging at different scales of spatial resolution and penetration depth. We demonstrated that the FRET‐based caspase‐3 biosensor enabled visualization of drug‐induced apoptosis in single cells at optical resolution using both PAM (3 µm) and fluorescence microscopy (resolution limited by diffraction, ≈350 nm). We also monitored apoptosis in brain tumors (>3 mm beneath the scalp) in vivo at 150‐µm resolution using PACT.

## 2 Results

### 2.1 Characterization of a NIR FRET Biosensor

**Figure 1:**
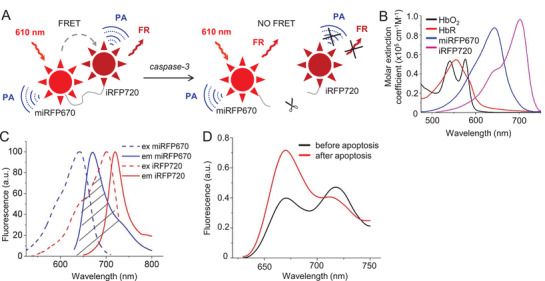
Characterization of a NIR miRFP670‐iRFP720 FRET caspase‐3 biosensor and PA imaging setup. A) Schematic design of FRET miRFP670‐iRFP720 caspase‐3 biosensor that is activated during apoptosis. Activated caspase‐3 cleaves the ‐DEVD‐ linker and releases miRFP670 and iRFP720 from each other, thus decreasing FRET. PA: photoacoustic signal. FR: fluorescence. B) Overlay of molar extinction spectra of oxyhemoglobin (HbO2), deoxyhemoglobin (HbR), miRFP670, and iRFP720. C) Excitation and emission spectra for miRFP670 and iRFP720. Dashed area indicates spectral overlap between the emission spectrum of miRFP670 (donor) and excitation spectrum of iRFP720 (acceptor). D) Spectral changes of miRFP670‐iRFP720 caspase‐3 biosensor measured in the suspension of HeLa cells stably expressing biosensor before (black line) and 3 h after (red line) staurosporine (STS)‐induced apoptosis. The spectra were normalized to the fluorescence intensity of iRFP720 excited at 700 nm, which does not change during apoptosis.

To explore NIR FRET with PAT, we developed an improved FRET biosensor for caspase‐3 based on miRFP670 donor and iRFP720 acceptor separated by the caspase‐3 cleavage site (**Figure** **1A**). We chose to work with the caspase‐3 biosensor, because this type of biosensors has been extensively characterized and provides robust responses in single cells. Thus, it perfectly suits the need to test the performance of the novel technology. The optical absorption spectra of miRFP670 and iRFP720 are red‐shifted relative to hemoglobin (Figure 1B). The donor miRFP670^[^ ^36^ ^]^ is spectrally similar to the previously developed dimeric iRFP670^[^ ^35^ ^]^ that was successfully used in PAT. The miRFP670‐iRFP720 FRET pair is characterized by a substantial spectral overlap between the donor fluorescence and acceptor excitation spectra (Figure 1C). The miRFP670‐iRFP720 caspase‐3 biosensor provided the highest response to cleavage in the suspension of mammalian cells. When excited at 610 nm, the fluorescence changes in the donor channel were almost twofold at the peak emission wavelength (Figure 1D). This is higher than that of the previously reported caspase‐3 biosensor containing a monomeric miRFP720 acceptor and having the reverse orientation of the donor and the acceptor in a fusion.^[^ ^37^ ^]^ According to the spectra (Figure 1D) obtained for the HeLa cells stably expressing the miRFP670‐iRFP720 biosensor, the donor/FRET ratio was 49%, compared to 34% reported for the biosensor with the miRFP720.^[^ ^37^ ^]^

Upon excitation, for the miRFP670‐iRFP720 caspase‐3 biosensor, the generated PA signal consists of the following Equation (1):

(1) $$PA_{1}=P_{\mathrm{DA}}+P_{A}+P_{i}$$

Here, *P*_DA_ is the PA signal generated by the donor miRFP670 in the presence of the acceptor iRFP720. *P*_DA_ is larger than *P*_D_, which is the PA signal generated by the donor in the absence of FRET, because the energy absorbed by the donor is transferred to the acceptor due to FRET.^[^ ^33^ ^]^ *P*_A_ is the direct PA signal generated by the acceptor iRFP720. In this work, we used 610‐nm light for FRET imaging. Although 610 nm is away from the donor excitation peak at 643 nm (Figure 1B), a use of 610 nm allows for minimizing background by reducing the direct absorption from the acceptor (iRFP720). *P_i_* is the PA signal from endogenous molecules at 610 nm. Once caspase‐3 cleaves the ‐DEVD‐ linker, miRFP670, and iRFP720 become separated from each other, resulting in no FRET. The PA signals can be expressed as:

(2) $$PA_{2}=P_{D}+P_{A}+P_{i}$$

By detecting the PA signal difference between *PA*_1_ and *PA*_2_, we can photoacoustically monitor changes in FRET.

### 2.2 Visualization of the Caspase‐3 Activity in Live Cells

**Figure 2:**
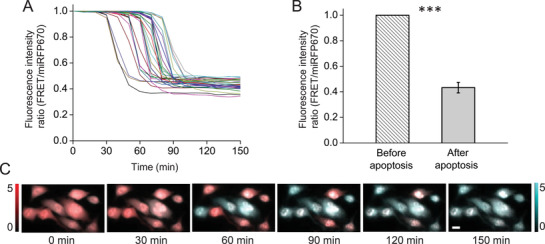
Fluorescence imaging of caspase‐3 activity in HeLa cells stably expressing miRFP670‐iRFP720 caspase‐3 biosensor during STS‐induced apoptosis. A) Kinetics of the fluorescence intensity ratio (FRET/miRFP670) for individual cells undergoing apoptosis. Each line represents one single cell. The images were taken every 10 min. B) Fluorescence intensity ratio (FRET/miRFP670) before and after STS‐induced apoptosis. *n* = 30 cells, error bar, s.d. ***, *p* < 0.001, calculated using a paired Student's *t*‐test. C) Representative fluorescence images in FRET (red) and miRFP670 (blue) channels at selected time points. Scale bars, 10 µm.

First, we tested the miRFP670‐iRFP720 biosensor, which was stably expressed in HeLa cells, using fluorescence microscopy. Upon addition of staurosporine (STS) to the cells, caspase‐3 activation resulted in biosensor responses (**Figure** **2**). The monitored fluorescence intensity ratio between the FRET (605/30 nm excitation and 725/40 nm emission filters) and the donor (605/30 nm excitation and 667/30 nm emission filters) channels decreased to the minimum of ≈40% in 30 min for individual cells and in less than 2 h for a population (Figure 2A,B). Fluorescence images (Figure 2C,D) illustrate the dynamics in individual cells at subcellular resolution.

**Figure 3:**
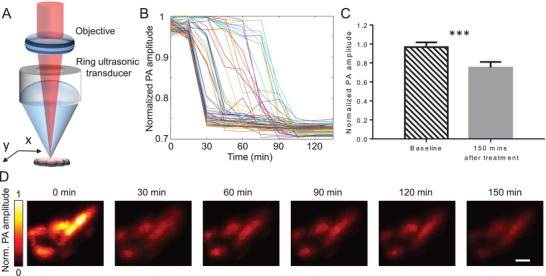
PAM of caspase‐3 activity in HeLa cells stably expressing miRFP670‐iRFP720 caspase‐3 biosensor during STS‐induced apoptosis. A) Setup of optical‐resolution PAM. B) PA amplitude changes for individual cells undergoing apoptosis. Each line represents one single cell. The images were taken every 10 min. C) PA amplitude before and after STS‐induced apoptosis. *n* = 40 cells, error bar, s.d. ***, *p* < 0.001, calculated using a paired Student's *t*‐test. D) Representative PAM images of cells at selected time points. Scale bars, 10 µm.

Next, we visualized the FRET sensor expressing cells in the same conditions using optical‐resolution PAM (**Figure** **3A**). Under 610‐nm illumination, PAM^[^ ^44^ ^]^ revealed the HeLa cells at a spatial resolution of ≈3–4 µm. After baseline imaging, STS was added to the cell culture media to induce apoptosis. The kinetics of the PA signals for individual cells (≈30 min) and the population (less than 2 h) were similar to those observed in fluorescence measurements (Figure 3B). After 120 min, the PA signals decreased to ≈70% of the baseline level (Figure 3C). PAM images also visualize the signal changes in individual HeLa cells during the apoptosis at subcellular resolution (Figure 3D).

### 2.3 Multiscale PAT of Caspase‐3 Activities In Vivo

**Figure 4:**
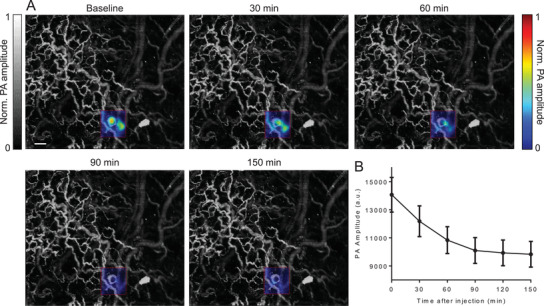
In vivo PAM of caspase‐3 activity in a mouse ear tumor. A) Representative PAM images of the mouse ear at selected time points. Pseudocolor shows the PA signals in the tumor area. Scale bars, 200 µm. The images were taken every 30 min. B) PA signals at the tumor region decrease after STS‐induced apoptosis. *n* = 3 mice, error bar, s.e.m.

We then carried out PA FRET imaging in vivo using both PAM and PACT. We first imaged the caspase‐3 activities in a mouse ear tumor using PAM at a single‐cell resolution. A xenograft tumor was induced in the mouse ear by the injection of 1 × 10^5^ HeLa cells expressing miRFP670‐iRFP720 caspase‐3 biosensor. Three days after injection, the mouse ear was imaged by PAM with 610‐nm illumination at a spatial resolution of ≈3–4 µm. The tumor and the ear vasculature (vessels are shown in gray and the tumor is shown in color) were clearly resolved, as shown in **Figure** **4A**. After baseline scanning, STS was injected into the tumor subcutaneously. We then monitored the PA signal changes in the tumor area. An obvious PA signal decrease was observed after the injection, as shown in Figure 4B, indicating the caspase‐3 activity during cell apoptosis.

**Figure 5:**
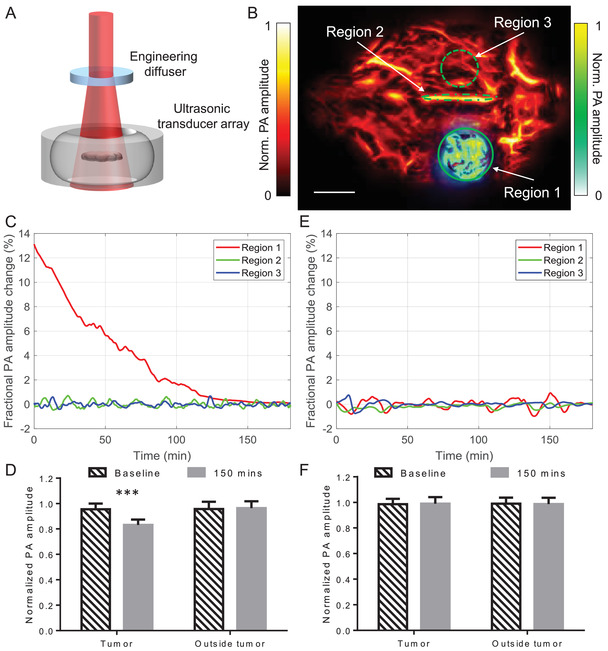
PACT of caspase‐3 activity in a mouse brain tumor expressing miRFP670‐iRFP720 caspase‐3 biosensor during STS‐induced apoptosis. A) Setup of PACT. B) PACT image of a mouse brain 2‐h post‐injection, where the tumor was highlighted by computing the difference from the baseline image. STS was injected locally into the tumor to induce apoptosis. Scale bar, 2 mm. The three regions are the tumor area on the left hemisphere (Region 1), the biggest vessel in the brain (superior sagittal sinus, Region 2), and the region contralateral to the tumor (Region 3). C) PA signal changes after STS‐induced apoptosis. PACT measurements were acquired at 1 Hz frame rate. D) Statistics of PA signals before and after treatment. *n* = 3 mice, error bars, s.e.m. ***, *p* < 0.001, calculated using a paired Student's *t*‐test. E) PA signal changes after PBS injection. PACT measurements were acquired at 1 Hz frame rate. F) Statistics of the control group. *n* = 3 mice, error bars, s.e.m.

To monitor the FRET processes in deep tissue, we imaged deep‐seated tumors in the brain using PACT^[^ ^9^ ^]^ (**Figure** **5A**). A tumor was induced in the mouse brain by the injection of 1 × 10^6^ HeLa cells expressing miRFP670‐iRFP720 caspase‐3 biosensor. Three weeks after injection, the mouse was imaged by PACT. During the in vivo experiments, the mouse was mounted onto a holder with the water bag placed on top, and ultrasound gel applied between the scalp and the water bag for ultrasonic coupling. To illuminate the whole brain, a broad laser beam set at 610 nm was used. The optical fluence on the scalp was 10 mJ cm^−2^. Throughout the experiments, the scalp was not removed. In the experimental group, we first obtained the baseline image of the tumor (3 mm beneath the scalp), we then injected STS (10 µL) in DMSO into the tumor to induce cell apoptosis. The mouse brain was monitored using PACT for ≈3 h. PACT image of a mouse brain 2‐h post‐injection is shown in Figure 5B, where the tumor was highlighted by computing the difference from the baseline image. A threshold level of four times the noise level, estimated as the standard deviation of the background signal outside the imaged region, was applied. We selected the three regions for monitoring of the signal changes: the tumor region, the contralateral region, and the biggest vessel in the brain (Figure 5B). PA signals of the tumor region decreased obviously, while PA signals from non‐tumor regions had no significant changes, which indicates the caspase‐3 activities inside tumor cells after the STS injection (Figure 5C,D). In the control group, we injected DMSO (10 µL) into the tumor and monitored the PA signal changes for ≈3 h post‐injection. Signals from three similar regions (as labeled in Figure 5B) were analyzed and are plotted in Figure 5E. No significant changes were observed either inside the tumor or in the normal tissue (Figure 5F).

## 3 Discussion

Here, we demonstrated multiscale PA imaging of FRET biosensors in living cells and live animals for the first time, to the best of our knowledge. We observed that fluorescence imaging of the same NIR biosensor in similar conditions correlated well with the PA imaging. Specifically, the kinetics of the biosensor responses in individual cells, the variability between cells, and responses in large cell populations (Figures 2A and 3B) were similar. Further, the kinetics of biosensor responses is similar for cell populations observed in vivo, showing the PA signals decreased to a plateau in 120–150 min after the STS stimulation (Figures 4B and 5C).

We explored the possibility of applying PAT at different resolutions and penetration depths in vivo. In both PAM and PACT, we distinguished vasculature based on hemoglobin contrast from tumor cells expressing the NIR FRET biosensor. PAM has monitored the FRET process at single‐cell resolution within 1‐mm in depth. PACT has visualized the FRET dynamic in deep seated tumors (>3 mm in depth) at 150‐µm spatial resolution. For fluorescence imaging, it is currently not possible to obtain anatomical and functional details at such resolution beyond 1 mm in depth.

We developed the NIR biosensor with the large dynamic range of FRET changes (donor/FRET ratio was 49% according to the spectra in Figure 1D), which allowed us to photoacoustically detect FRET changes in the presence of the background. The background absorption *P_i_* (Equations 1 and 2) originates from endogenous molecules, including heme, porphyrins, lipids, and melanin.^[^ ^34^, ^45^ ^]^ While hemoglobin is the main endogenous absorber in vivo, heme‐binding cytochromes significantly contribute to the background absorbance in cultured cells.^[^ ^46^ ^]^ We experimentally confirmed this by measuring PA signals from non‐transfected HeLa cells (Figure S1, Supporting Information). Absorption of all heme‐binding proteins drops significantly at wavelengths longer than 600 nm (Figure 1B; Figure S1, Supporting Information). In fact, absorption of heme‐binding proteins is orders of magnitude lower at 600 nm compared to 410–430 nm, which is required for widely used GFP‐like cyan‐yellow FRET biosensors. Therefore, the NIR biosensor and imaging at 610 nm wavelength allowed us to detect FRET changes over the background in cells and in vivo at depths.

To summarize, here we demonstrated that a NIR FRET biosensor with a large dynamic range of response allowed photoacoustic imaging of molecular processes in vivo. We suppose that the reported technology can be applied to imaging of other miRFP670‐(m)iRFP720 NIR biosensors, which are characterized by at least twofold changes in the donor channel. The limitation here is the magnitude of changes per pixel, which depends on the spatial resolution of the PA imaging systems (PAT resolution voxel size) and the total biosensor responses per single cell and/or cell population. The caspase‐3 biosensor that we used here reported a synchronous response in cells, enhancing the dynamic range in vivo. We speculate that the NIR calcium biosensor iGECI,^[^ ^41^ ^]^ which has sixfold changes in the donor channel and reports changes in a whole cell, should be applicable to the single‐cell PAM. When expressed in the heart muscle cells with synchronous responses,^[^ ^47^ ^]^ it should also be applicable to lower resolution but more penetrating PACT for the visualization of cardiac activities. The NIR biosensors with a low dynamic range of responses^[^ ^48^ ^]^ and low total changes per cell^[^ ^37^ ^]^ are not optimal for PAT. While more red‐shifted fluorescent proteins should decrease the background further, they have not been developed yet. A way around is the development of new biosensors with a photoswitchable donor. It should result in a further reduction of the background, improving the detection sensitivity and extending the non‐invasive photoacoustic in vivo FRET imaging to more biological processes in biology and biomedicine.

## 4 Experimental Section

### Optical‐Resolution PAM of Caspase‐3 Activities in the Mouse Ear

As shown in Figure 3A, a pulsed Nd:YAG laser (Innoslab, 532 nm, Edgewave, 2‐kHz repetition rate) pumps a dye laser (CBR‐D, Sirah) to provide 610‐nm light for PA excitation. The illumination beam was focused by an objective (AC127‐050‐A, Thorlabs; NA, 0.1 in air) through a ring‐transducer into the mouse ear from the top. The resultant PA waves were detected by a ring‐shape ultrasonic transducer (35 MHz center frequency, 25 MHz bandwidth, from the University of Southern California). The ring‐transducer had a 2 mm diameter hole in the center for the optical illumination beam to pass through. It had 6‐mm working distance and an acoustic NA of 0.3. Volumetric imaging was acquired by 2D raster scanning of the mouse ear. The PAM system provides a spatial resolution of ≈3–4 µm with a penetration depth of 1 mm. For PAM imaging of cells in culture, it took 20 s to finish one PAM image, and images every 10 min were acquired. For PAM imaging of tumors in the mouse ear, it took 5 min to finish one PAM image, and images every 30 min were acquired.

### PACT of Caspase‐3 Activities in the Mouse Brain

The PACT system used in this study is shown in Figure 5A. A lab‐made optical parametric oscillator (OPO) laser, pumped by an Nd:YAG laser with a 10‐Hz pulse‐repetition rate, was used to output 610 nm for PA excitation. The laser beam was first homogenized by an optical diffuser (EDC‐5, RPC Photonics) and then illuminated the mouse brain from the top. The maximum light fluence on the skin of the animal was ≈10 mJ cm^−2^, which was well below the American National Standards Institute safety limit. The PA signals were detected by a full‐ring ultrasonic transducer array (Imasonic) with a 10‐cm diameter, a 5‐MHz central frequency, more than a 90% one‐way bandwidth, and 512 elements. Each element (20‐mm height, 0.61‐mm pitch, and 0.1‐mm inter‐element space) was cylindrically focused to produce an axial focal distance of 45 mm (acoustic NA, 0.2). The combined foci of all 512 elements form an approximately uniform imaging region with a 20‐mm diameter and 1‐mm thickness. In this region, the in‐plane spatial resolution was ≈150 µm. A lab‐made 512‐channel preamplifier (26 dB gain) was directly connected to the ultrasonic transducer array housing, with minimized connection cable length to reduce cable noise. The pre‐amplified photoacoustic signals were digitized using a 512‐channel data acquisition system (four SonixDAQs, Ultrasonix Medical ULC; 128 channels each; 40‐MHz sampling rate; 12‐bit dynamic range) with programmable amplification up to 51 dB. The data acquisition time for each frame was 50 µs. Each laser pulse yields a widefield image and the frame rate was 10 Hz, currently limited by the laser repetition rate. The digitized radio frequency data were first stored in the onboard buffer, then transferred to a computer. The digitized raw data were fed into a half‐time dual‐speed‐of‐sound universal back‐projection algorithm for image reconstruction.

### FRET Biosensor Development

To obtain miRFP670‐iRFP720 caspase‐3 biosensor, iRFP720 acceptor was inserted instead of miRFP720, and the fluorescent proteins miRFP670 and iRFP720 were swapped, compared to the construct reported in Ref. ^[^ ^37^ ^]^. The linker between two proteins was GGDEVDGPVAT and the plasmid was obtained from pEGFP‐N1 plasmid (Clontech).

### Mammalian Cell Culture

HeLa cells were grown in DMEM medium supplemented with 10% FBS supplemented with penicillin‐streptomycin (all from Life Technologies‐Invitrogen) at 37 °C in 5% CO_2_. Transient cell transfections were performed using an Effectene reagent (Qiagen). Preclonal mixtures of HeLa cells were obtained using selection on 700 µg mL^−1^ of G418 antibiotic for 2 weeks and enriched with FACSAria (BD Biosciences) fluorescence‐activated cell sorter using 640‐nm light for excitation.

### Sensor Characterization in Mammalian Cells

For characterization in cell suspension, 2–10 µm staurosporine (STS) was added to cells grown in 6‐well plates for 6 h before the analysis. Fluorescence spectra of the untreated (uncleaved) and staurosporine‐treated (cleaved) cell suspensions were recorded using 610 nm excitation and normalized by acceptor fluorescence at 670 nm for comparison.

For fluorescence microscopy, cells were cultured in 35‐mm glass‐bottom Petri dishes with no. 1 coverglass (MatTek). Live HeLa cells were imaged on Olympus IX81 inverted epifluorescence microscope operated with SlideBook v.4.1 software (Intelligent Imaging Innovations) and equipped with a 60  ×  1.35 numerical aperture (NA) oil objective lens (UPlanSApo, Olympus) and an opiMOS sCMOS camera (QImaging). During imaging, HeLa cells were incubated in a cell imaging medium (Life Technologies‐Invitrogen) and kept at 37 °C. For FRET, a 605/30 excitation filter and two emission filters (667/30 nm for miRFP670 and 725/40 nm for iRFP720) were used. Fluorescence intensity ratio between FRET and donor was calculated. FRET measurements were quantified using ImageJ (NIH). Time‐course ratio measurements were normalized to baseline prestimulation values.

### Animal Preparation

Adult, 2–3‐month‐old female nude mice (Hsd:Athymic Nude‐FoxlNU, JAX.; body weight, ≈20−30 g) were used for all in vivo experiments. All experimental procedures were carried out in conformity with laboratory animal protocol (IA20‐1737) approved by the Institutional Animal Care and Use Committee of California Institute of Technology. During the OR‐PAM experiments, the mouse was taped to a lab‐made motorized animal holder, and one of its ears was attached to an ear plate for high‐resolution imaging. During the PACT experiments, the mouse was taped to a lab‐made animal holder so that its cortex was aligned with the acoustic focal plane of the transducer array for imaging. The mouse was then placed under the water bag of the imaging system, and ultrasound gel was applied between the head and the water bag for ultrasonic coupling. The holder was then lifted until the tumor was in the focal plane of the transducer array. During the PACT in vivo experiment, the authors did not remove the scalp. The STS was administrated via a needle injection through the scalp. The injection procedure was guided by PACT. The tumor injection process was recorded initially. During STS injection, PACT could real‐time visualize the needle injection. And the injection started when the needle tip reached the tumor region. Throughout the experiment, the mouse was maintained under anesthesia with 1.5% vaporized isoflurane and the animal body temperature was regulated toward 38 °C.

### Statistical Analysis

For fluorescence quantification, the background that corresponds to the mean signal from cell‐free areas was subtracted. A threshold‐based segmentation method was applied to identify cells. The average signals in each cell were used in statistical analysis and to plot fluorescence intensity ratios (FRET/miRFP670) in Figure 2A. For in vitro photoacoustic quantification in cells, a threshold level of eight times the noise level was applied and each cell was identified. The PA signals within each cell were averaged to represent every single cell, plotted in Figure 3B and used in statistical analysis. For in vitro experiments, the data were presented as mean values ± standard deviations (SDs). For the PA measurements in vivo, differential operations were applied between the baseline image and after‐injection image; then, a threshold level of four times the noise level, estimated as the standard deviation of the background signal outside the imaged region, was applied as in Figures 4A and 5B. The authors used the average pixel values in the differential images for statistical analysis. For in vivo experiments, the data were presented as mean values ± standard errors of the mean (s.e.m.). Sample sizes were *N* = 30 cells for fluorescence, *N* = 40 cells for photoacoustic quantifications in vitro, and *N* = 3 mice for both PAM and PACT quantifications in vivo. The statistical significance of a difference between two groups was calculated using a paired Student's *t*‐test. *P* value of *p* < 0.001 was set as a significance threshold in all tests. Statistical analysis and data processing were carried out using Origin (OriginLab) software for fluorescence and MATLAB (MathWorks) for photoacoustic quantifications.

## Conflict of Interest

L.V.W. has financial interests in Microphotoacoustics, Inc., CalPACT, LLC, and Union Photoacoustic Technologies, Ltd., which did not support this work. The other authors declare no competing interests.

## Author Contributions

L.L. and H. H. characterized the biosensor in photoacoustic experiments. D.M.S. developed the caspase‐3 sensor and characterized it in mammalian cells using fluorescence. V.V.V., L.W., and D.M.S. directed the project. L.L. and D.M.S. wrote the manuscript with contributions from all authors.

## Supporting information

Supporting Information

## Data Availability

The data that support the findings of this study are available from the corresponding author upon reasonable request.
