# Supplementary material for: Multiscale Photoacoustic Tomography of a Genetically Encoded Near‐Infrared FRET Biosensor
Source: Adv Sci (Weinh). 2021 Sep 17;8(21):2102474. doi: 10.1002/advs.202102474 (PMC8564460; doi:10.1002/advs.202102474)
Supplement: Supplementary file 1 — Supporting Information [file ADVS-8-2102474-s001.pdf]

© 2021 Wiley-VCH GmbH

ADVANCED SCIENCE
Open Access

Supporting Information

for *Adv. Sci.,* DOI: 10.1002/advs.202102474

Multiscale photoacoustic tomography of a genetically encoded near-infrared FRET biosensor

Lei Li, Hsun-Chia Hsu, Vladislav V. Verkhusha, Lihong V. Wang, and Daria M. Shcherbakova**

Multiscale photoacoustic tomography of a genetically encoded near-infrared FRET biosensor

Lei Li, Hsun-Chia Hsu, Vladislav V. Verkhusha, Lihong V. Wang, and Daria M. Shcherbakova

Supplementary Information

Supplementary Figure 1. The background is generated by heme-binding proteins in cultured cells.

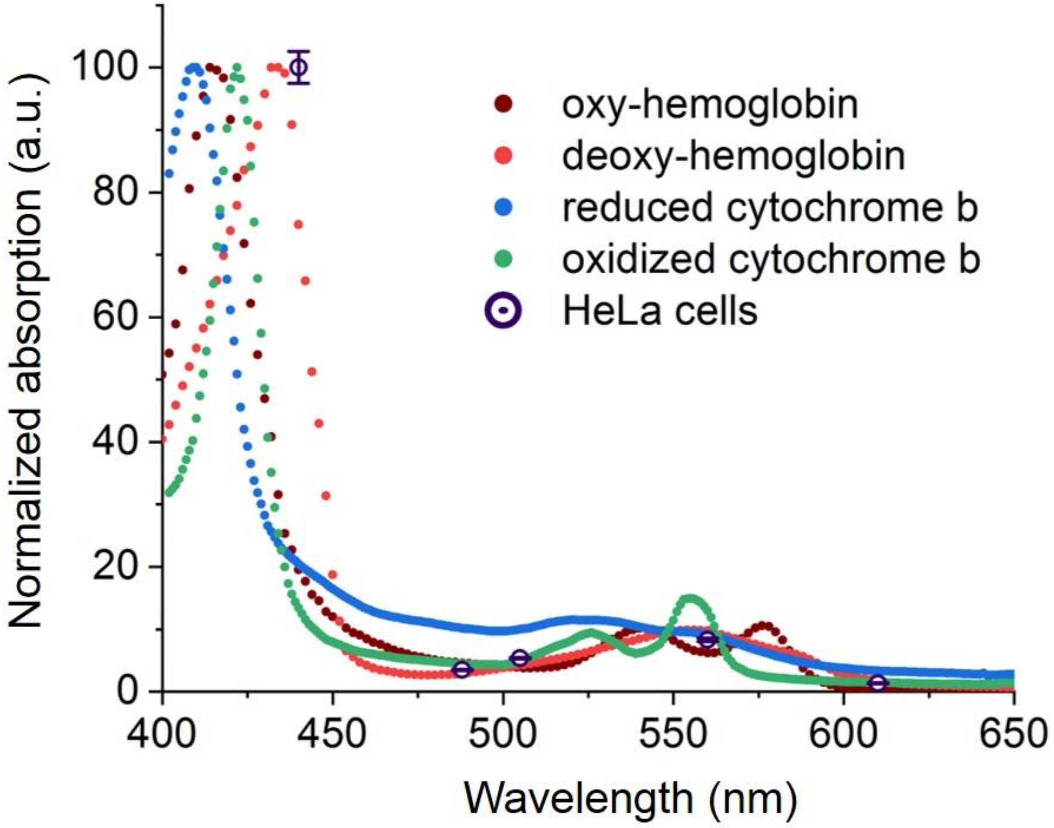

Normalized absorbance spectra of heme-binding proteins (hemoglobin and cytochrome b) and normalized photoacoustic signals of HeLa cells were overlayed. The photoacoustic signal is directly proportional to the absorption of molecules. Several cytochrome proteins are found in mammalian cells: cytochromes a, a3, b, and c [45]. We present here the spectra of the most abundant cytochrome b (43% of cytochrome b, 21% cytochrome c, and 36% of more red-shifted cytochrome a,a3). The signals from non-transfected HeLa cells *in vitro* were imaged at different wavelengths using the PACT (setup described in the Main text). Mean values and SEM were normalized and plotted.
